# Einführung des Fachs „Berufsfelderkundung“ für Studierende der Zahnmedizin an der Universität des Saarlandes

**DOI:** 10.1007/s00103-023-03790-5

**Published:** 2023-11-07

**Authors:** Stefan Rupf, Madline Gund, Ulf Strähle, Jusef Naim, Matthias Hannig

**Affiliations:** 1 Professur für Synoptische Zahnmedizin, Universität des Saarlandes, Homburg, Saarland Deutschland; 2 Klinik für Zahnerhaltung, Parodontologie und Präventive Zahnheilkunde, Universität des Saarlandes, Homburg, Saarland Deutschland; 3 Professur für Synoptische Zahnmedizin, Universität des Saarlandes, Gebäude 73, Kirrberger Str. 100, 66421 Homburg/Saar, Deutschland

**Keywords:** ZApprO, vorklinisches Studium, Kompetenzen, Curriculumsentwicklung, Evaluation, ZApprO, Preclinical studies, Skills, Curriculum development, Evaluation

## Abstract

Seit dem Wintersemester 2021/2022 gilt die neue zahnärztliche Approbationsordnung (ZApprO). Darin wurde festgelegt, dass ein „Praktikum der Berufsfelderkundung“ als Lehrveranstaltung im Zahnmedizinstudium durchgeführt wird. In der Universität des Saarlandes besteht die Lehrveranstaltung aus 5 Teilen: (I) Einführung, (II) Praktikum, (III) Reflexionsbericht, (IV) Symposium, (V) Evaluation. Das Praktikum soll den Studierenden eine frühe Berührung mit der klinischen Realität ermöglichen und ihnen mehr Selbstvertrauen im Umgang mit Patienten geben, sie motivieren und ihnen die professionelle Rolle im Gesundheitsberuf vermitteln. Zusätzlich soll durch den Reflexionsbericht und das Symposium die Entwicklung wissenschaftlicher Kompetenzen initiiert werden. Die Evaluation dient zusammen mit der Bewertung der Reflexionsberichte der Überprüfung des Erfolgs der Einführung des Faches. Ziel dieses Beitrags ist es, die neue Lehrveranstaltung zu beschreiben und die Ergebnisse der Auswertung der beiden Veranstaltungen in den Jahren 2021 und 2022 darzustellen.

## Abstract (en)

The new dental licensing regulations (ZApprO) have been in effect since the 2021/2022 winter semester and stipulate that a “professional field exploration course” be offered in the dentistry degree program. At Saarland University, the course consists of five parts: (I) introduction, (II) practicum, (III) reflection report, (IV) symposium, and (V) evaluation. The practicum is designed to provide students with earlier exposure to clinical reality, give them more confidence in dealing with patients, motivate them, and teach them about the professional role in the healthcare profession. In addition, the reflection report and symposium will initiate the development of scientific competencies. The evaluation, together with the evaluation of the reflection reports, serves to verify the success of the introduction of the course. The purpose of this paper is to describe the new course and to present the results of the result of the evaluation of the two courses in 2021 and 2022.

## Einleitung

Die im Regelstudiengang Zahnmedizin im Wintersemester 2021/2022 neu eingeführte Approbationsordnung für Zahnärzte und Zahnärztinnen (ZApprO; [1]) beschreibt als Zielstellung für die zahnärztliche Ausbildung „den Zahnarzt und die Zahnärztin, der oder die wissenschaftlich und praktisch in der Zahnmedizin ausgebildet und zur eigenverantwortlichen und selbständigen Ausübung der Zahnheilkunde, zur Weiterbildung und zu ständiger Fortbildung befähigt ist.“ Insbesondere intra- und interdisziplinäre Zusammenarbeit mit der Medizin und Angehörigen anderer Berufe des Gesundheitswesens werden in den Vordergrund gestellt [1]. Mit der übergeordneten Strukturierung in 3 Ausbildungsabschnitte und der Einführung von interdisziplinären Querschnittsbereichen wird die ZApprO an die Approbationsordnung für Ärzte (ÄApprO; [2]) angenähert. Sie öffnet und fordert gleichzeitig den Weg zur longitudinalen Vermittlung klinischen Wissens und erfordert eine fächerübergreifende Betrachtung der Wissensvermittlung.

Im ersten Ausbildungsabschnitt werden gemäß ZApprO die naturwissenschaftlichen Fächer und die klassischen Fächer der Vorklinik angeboten. Ein bedeutender Unterschied zur Vorgängerverordnung ist allerdings, dass die Grundlagenfächer des ersten Abschnitts zahnmedizinisch bedeutsame und insbesondere klinische Zusammenhänge vermitteln und prüfen sollen (§ 32 [1]). Die vorklinische zahnmedizinische Ausbildung stützt sich auf das Fach „Zahnmedizinische Propädeutik“, das dentaltechnologische und präventivzahnmedizinische Teile enthält. Zusätzlich wurde ein „Praktikum der Berufsfelderkundung“ in die zahnmedizinische Ausbildung eingeführt, für welches die regelmäßige und erfolgreiche Teilnahme für die Zulassung zum ersten Abschnitt der zahnärztlichen Prüfung nachgewiesen werden muss. Im Zusammenhang mit dem Praktikum findet jedoch keine Prüfung statt. Es soll die frühe Berührung mit der klinischen Realität in Form einer Hospitation ermöglichen, u. a. um den Studierenden mehr Selbstvertrauen im Umgang mit Patienten zu geben, sie zu motivieren und ihnen die professionelle Rolle im Gesundheitsberuf zu vermitteln [3].

Es liegen nur wenige Publikationen aus Deutschland zu diesem Fach im Rahmen der Ausbildung der Studierenden der Medizin vor [4, 5]. Eine Studie legte Studierenden 4 unterschiedliche Konzepte zur Bewertung vor und stellte fest, dass die Möglichkeit, sich einen Überblick über verschiedene Arbeitsfelder in Form mehrerer Hospitationen zu verschaffen, von den Studierenden präferiert wird. Vorlesungsbasierte Konzepte sowie solche mit Schwerpunkten auf Kernkompetenzen wie Kommunikation oder dem Umgang mit chronischem Kranksein wurden schlechter beurteilt [6].

An der medizinischen Fakultät der Universität des Saarlandes wurde entsprechend der neuen Approbationsordnung das Fach „Berufsfelderkundung“ im Wintersemester 2021/2022 ins Curriculum der zahnmedizinischen Ausbildung integriert. Es findet als Lehrveranstaltung „Berufsfelderkundung“ im 1. Semester statt, zeitlich vor den Praktika der Zahnmedizinischen Propädeutik. Das Fach Berufsfelderkundung ist in 5 Abschnitte unterteilt: (I) Einführung, (II) Praktikum, (III) Reflexionsbericht, (IV) Symposium, (V) Evaluation. Insgesamt soll das Fach den Studierenden ermöglichen zahnmedizinische Versorgungsstrukturen kennenzulernen, ein grundlegendes Verständnis des Berufsbildes zu erlangen sowie kommunikative und wissenschaftliche Kompetenzen zu entwickeln. Die Studierenden werden auf ihre Hospitation in Einrichtungen des zahnmedizinischen Berufsfeldes vorbereitet und erhalten konkrete Aufträge, die sie während ihrer Zeit in den Einrichtungen bearbeiten sollten. Nach Abschluss der Hospitation werden die Studierenden gebeten, ihre Erlebnisse in einem an die Struktur wissenschaftlicher Publikationen angelehnten Reflexionsbericht zu beschreiben. Zusätzlich wird der Auftrag erteilt, in Gruppenarbeit interessante Themenfelder, die während der Hospitation deutlich werden, zu bearbeiten und in einem Symposium darzustellen.

Die primäre Zielstellung ist es, den Studierenden die Möglichkeit zu geben, das Berufsbild der Zahnmedizin zu erkunden, indem Zahnmedizinerinnen und Zahnmediziner beobachtet und interviewt werden, um die professionellen Rollen, die von Zahnärztinnen und Zahnärzten eingenommen werden [7], zu reflektieren. Durch den frühen Kontakt mit zahnmedizinischen Teams zu Beginn des Studiums wird angestrebt, die Motivation für das Studium und die professionelle Haltung zu fördern [8].

Die Überprüfung des Erfolgs der Einführung des Faches erfolgte mithilfe der Bewertung der Reflexionsberichte und der Evaluation aus den Jahren 2021 und 2022. Sie soll in dem vorliegenden Beitrag neben der Beschreibung der neuen Lehrveranstaltung dargestellt werden.

## Umsetzung des Fachs „Berufsfelderkundung“ 2021 und 2022

### Studierende

Die Anzahl der teilnehmenden Studierenden im Jahr 2021 betrug 26 Personen, im Jahr 2022 startete die Veranstaltung mit 25 Teilnehmerinnen und Teilnehmern.

### Aufbau der Lehrveranstaltung

**Figure 1:**
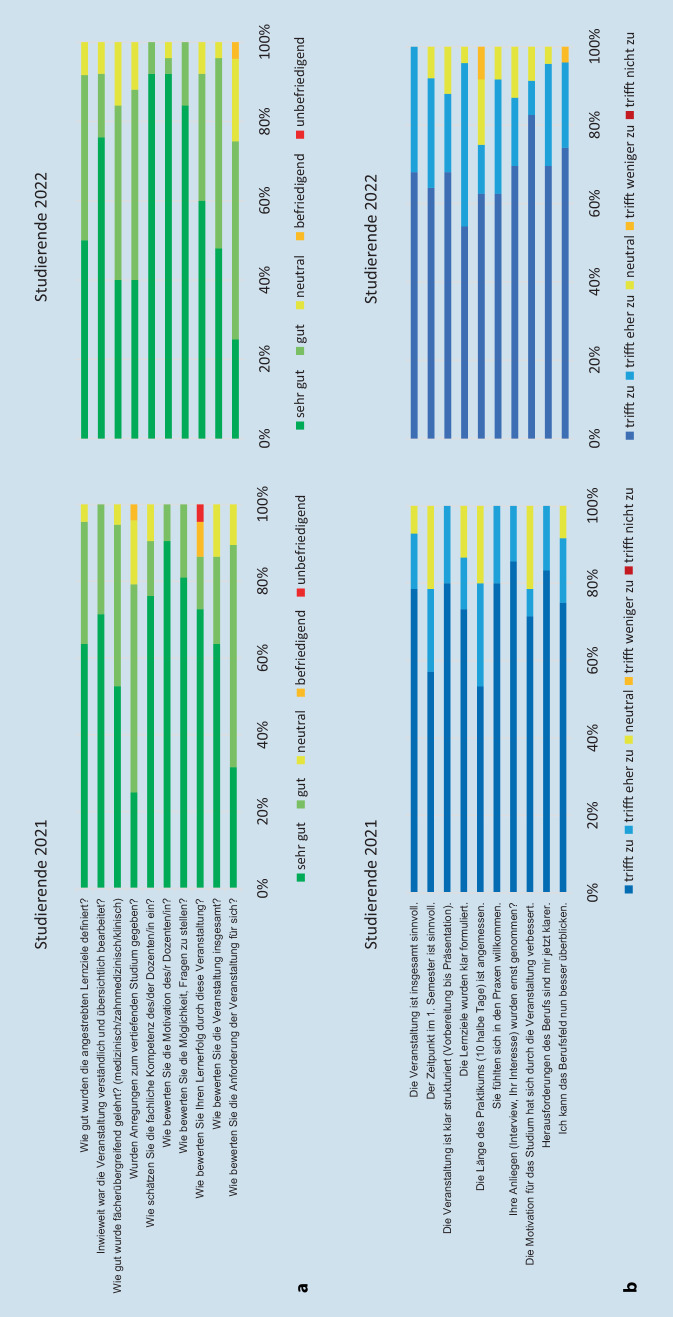

Das Fach Berufsfelderkundung wurde in 5 Abschnitte untergliedert:

1. *Einführung.* In den ersten Semesterwochen erfolgte an 4 Terminen wöchentlich eine 60-minütige thematische Einführung in das Fach. Es wurden den Studierenden die Ziele des Praktikums der Berufsfelderkundung dargestellt, eine grundlegende Einführung in die Zahnmedizin im Format einer Vorlesung präsentiert, das zahnmedizinische Berufsbild durch niedergelassene Kolleginnen und Kollegen seminaristisch dargestellt und eine Verteilung von zahnmedizinischen Einrichtungen vorgenommen, die sich im Vorfeld bereit erklärt hatten, Hospitanten im Rahmen der Berufsfelderkundung aufzunehmen. Wünsche der Studierenden bei der Einrichtungswahl wurden berücksichtigt, sofern es sich nicht um elterliche oder verwandtschaftliche Einrichtungen oder die Praxis des Hauszahnarztes bzw. der Hauszahnärztin handelte.
2. *Praktikum.* Das eigentliche Praktikum (Hospitation) erfolgte über einen Zeitraum von 42 h. Es sollten mindestens 2 Einrichtungen der zahnmedizinischen Versorgung frequentiert werden, wobei hier vor allem Kliniken und Polikliniken mit Ausnahme von Universitätskliniken, Praxen der ambulanten zahnmedizinischen Versorgung und Zahntechniklabore besucht werden sollten. Pflegeeinrichtungen, der Medizinische Dienst der Krankenkassen, Einrichtungen der Selbstverwaltung und Zahnärztekammern konnten tageweise mit einbezogen werden. Aufgrund der Lage des Saarlandes in der Großregion Saar-Lor-Lux waren auch Besuche in ausländischen Praxen möglich.
3. *Reflexionsbericht.* Die Erstellung eines Reflexionsberichts sollte in vorgegebener Form und Struktur zu einem Interview und zu den professionellen Rollen von Zahnärztinnen und Zahnärzten erfolgen. Nach Informationen zum Hintergrund, wie dem Zeitpunkt, an dem die Einrichtungen aufgesucht wurden, sollte ohne Namensnennung eine kurze Charakterisierung der Einrichtung erfolgen, dann sollten die Profile der Praxen unter Berücksichtigung der Lage (städtisch/ländlich) beschrieben sowie die Anzahl der Beschäftigten und die Aufgabenbereiche der Mitarbeiterinnen und Mitarbeiter dargestellt werden. Die Ergebnisse zur Abbildung der professionellen Rollen und zum Interview sollten diskutiert werden. Abschließend sollte dargestellt werden, inwiefern die Studierenden von der Hospitation profitiert haben und wie sie ihr Wissen zum Berufsbild erweitern konnten. Die Studierenden erhielten durch den Verantwortlichen der Lehrveranstaltung (S.R.) ein kurzes Feedback zu ihrem Reflexionsbericht. Im Jahr 2022 wurden den Studierenden als Intervention zur Verbesserung des Outcomes Best-Practice-Beispiele für den Reflexionsbericht zur Verfügung gestellt.
4. *Selbstständige Durchführung eines Symposiums.* Der Zeitpunkt wurde gemeinsam mit den Studierenden festgelegt. Die Plattform Teams (Microsoft Corp., Redmond, USA) wurde durch die Universität des Saarlandes bereitgestellt. Die Einladung der Praxen, die Studierende aufgenommen hatten, erfolgte durch den Verantwortlichen der Lehrveranstaltung. Die Themen wurden gemeinsam festgelegt. Die Gruppen zur Bearbeitung der Themen mit 4–6 Personen organisierten die Studierenden selbst und die Moderation erfolgte durch die Studierenden mit Unterstützung durch den Verantwortlichen. Die Präsentationen sollten jeweils 15 min dauern und konnten entweder von einem oder mehreren Studierenden gehalten werden. Die Studierenden erhielten durch den Verantwortlichen eine verbale Einschätzung ihrer Präsentation während des Online-Symposiums.
5. *Evaluation.* Die Evaluation bestand aus 2 Teilen. Im ersten Teil wurden 10 Items zur Fakultät abgefragt, mit einem Fragebogen, der für jede curriculare Lehrveranstaltung im Bereich Medizin verwendet wird. Der zweite Teil enthielt 10 selbst entworfene Items, die auf das Fach Berufsfelderkundung fokussiert waren sowie Raum für Freitextanmerkungen boten (Abb. 1). Die Evaluation erfolgte vollständig anonym und papiergebunden.

### Identifikation von Einrichtungen

In Vorbereitung der Einführung des Faches wurden im Sommer des Jahres 2021 Informationsveranstaltungen durchgeführt, die sich an niedergelassene Zahnmedizinerinnen und Zahnmediziner richteten. Die Einladung erfolgte durch die Ärztekammer des Saarlandes, Abteilung Zahnärzte. Es nahmen insgesamt 63 Praxen an diesen Veranstaltungen teil, von denen 44 ihre Bereitschaft zur Teilnahme bekundeten und sich mit einer Weitergabe ihrer Adressen einverstanden erklärten. Die Praxisinhaber wurden gebeten, die Hospitation ihrer Praxisversicherung zu melden. Die Universität des Saarlandes stellte den vollständigen Impfstatus für SARS-CoV‑2 und die Masern sicher.

### Aufgaben der Studierenden während der Hospitation in den Einrichtungen

Das übergeordnete Ziel der Veranstaltung bestand darin, zahnmedizinische Berufsfelder kennenzulernen und die für die Patientenversorgung notwendigen Strukturen und Kompetenzen der handelnden Personen zu reflektieren. Dazu sollten die Studierenden als erste Aufgabe bei der Hospitation den in den Einrichtungen verantwortlichen Personen, in der Regel Zahnärztinnen und Zahnärzte, im Sinne eines Interviews folgende Fragen stellen:

- Welche Gründe führten zur Wahl des Berufs?
- Wie lange dauerte die Aus- und Fortbildung?
- Wie groß ist der Aufwand für Weiterbildung und Standespolitik?
- Wie ist das Verhältnis von Arbeitszeit, Entgelt und persönlicher Zufriedenheit?
- Wie schätzen die Zahnmediziner die momentane Situation des Berufsstandes ein?

In der zweiten Aufgabe sollten die Studierenden die Rollenbilder des Berufes, die im Nationalen Kompetenzbasierten Lernzielkatalog (NKLZ) dargestellt sind, beobachten und reflektieren (Tab. 1).

**Table 1:** 

Rollenbilder von Zahnärztinnen und Zahnärzten (nach NKLZ)	Fragen und Aufgabenstellungen
Medizinische Experten	Welcher Teil des Berufsfeldes bzw. welches Tätigkeitsspektrum wurde abgedeckt?
	War eine Spezialisierung vorhanden?
	Welche Krankheitsbilder wurden vorrangig behandelt?
Gelehrte	Gab es Ihnen bisher unbekannte Fachbegriffe? Bitten Sie die Zahnmediziner, Ihnen diese zu erklären, und notieren Sie 3 Beispiele, die Sie nun kennen!
	Welche Unterschiede gibt es zwischen Assistenz und Zahnmediziner bzw. Zahnmedizinerin?
Kommunikatoren	Sind die Zahnmediziner spezifisch auf unterschiedliche Patientengruppen eingegangen? Führen Sie ein Beispiel an!
	Wie würden Sie die Atmosphäre in der Praxis (anonymisiert) beschreiben?
Mitglieder eines Teams	Wie groß war das Praxisteam?
	Wie war die Hierarchie strukturiert?
	Wurde Teamentwicklung betrieben?
Gesundheitsberater und -fürsprecher	Haben die Zahnmediziner auf die Mundgesundheit der Patienten eingewirkt?
	Gab es Initiativen der Zahnmediziner gegenüber Behörden oder Versicherungen zugunsten der Patienten? Beschreiben Sie!
Verantwortungsträger und Manager	Vergleichen Sie, wenn möglich, Verantwortlichkeiten von Praxisinhabern und Zahnärzten im Angestelltenverhältnis oder des Assistenzpersonals!
Professionell Handelnde	Wie war der Tag organisiert?
	Welches waren die häufigsten Behandlungen der Praxis?
	Bitte reflektieren Sie, ob ethische Aspekte Teil der Therapieentscheidungen waren!

### Evaluation und Bewertung der Reflexionsberichte

Für die Evaluationsbögen wurden 5‑stufige Likert-Skalen verwendet – Evaluation der Fakultät: 1 („sehr gut“) bis 5 („unbefriedigend“); fachbezogene Fragen: 1 („trifft zu“) bis 5 („trifft nicht zu“). Von den einzelnen Items und dem Gesamtscore wurden Mittelwerte gebildet. Für beide Teile der Evaluation wurde jeweils Cronbachs Alpha ermittelt [9, 10], um deren interne Konsistenz abzuschätzen.

Die Bewertung des Reflexionsberichts erfolgte durch 2 Personen (S.R., J.N.). Es wurden maximal 10 Punkte für den Inhalt (Bearbeitung der 2 Aufgabenstellungen), 5 Punkte für Struktur und Stil (Gliederung in Abschnitte mit Zwischenüberschriften, anonyme Darstellung, Beschreibungsteil, Diskussionsteil vorhanden, verständliche Ausdrucksweise) und 5 Punkte für die Einhaltung formaler Vorgaben (Textlänge 2–3 Seiten, Schriftgröße, Zeilenabstand, einheitliche Formatierung, Rechtschreibung/Grammatik) vergeben. Auf Basis der Gesamtpunktzahl wurde für jeden Teilnehmenden ein Score-Wert ermittelt. Es wurde eine 5‑stufige Bewertungsskala mit einheitlichen Intervallen genutzt (Score 1: 17–20, Score 2: 13–16, Score 3: 9–12, Score 4: 5–8 Punkte und Score 5: ≤ 4 Punkte). Für die beiden Studierendengruppen wurde jeweils ein mittlerer Score-Wert errechnet. Der Vergleich zwischen den Gruppen 2021 und 2022 erfolgte mit dem Mann-Whitney-U-Test.

## Auswertungen zum Fach „Berufsfelderkundung“ 2021 und 2022

### Besuchte Einrichtungen

Die Hospitationen erfolgten zu freien Zeiten in der Vorlesungszeit und teilweise in den Ferien zum Jahreswechsel. Die Studierenden besuchten vor allem Zahnarztpraxen und zahnmedizinische Kliniken. Zahntechnische Einrichtungen wurden im Rahmen der Praxisbesuche tageweise aufgesucht.

Von den 44 saarländischen Einrichtungen, die sich nach dem Aufruf der Ärztekammer des Saarlandes, Abteilung Zahnärzte, bereit erklärt hatten, wurden von den 2 Jahrgängen 36 Praxen und 1 Klinik besucht. 3 Praxen zogen ihr Einverständnis aus persönlichen Gründen zurück, in 4 Praxen traten zum geplanten Hospitationszeitpunkt SARS-CoV-2-Infektionen auf, sodass Alternativen genutzt wurden. Es wurden außerhalb des Saarlandes zusätzlich Praxen in Sachsen, Rheinland-Pfalz, Schleswig-Holstein, in Luxemburg und Frankreich sowie eine Poliklinik und eine Praxis in Baden-Württemberg aufgesucht.

Bei der Zuordnung der Einrichtungen im Vorfeld der Hospitation fiel auf, dass solche am jeweiligen Heimatort der Studierenden und in der Region Homburg/Saarbrücken bevorzugt wurden. Die Einrichtungen am Heimatort wurden hinsichtlich familiärer Beziehungen kritisch hinterfragt. Da die Studierenden diese Einrichtungen zudem häufig nur zum Jahreswechsel erreichen konnten, fielen diese aufgrund von Urlaub jedoch in den meisten Fällen aus. Um einzelne Praxen nicht durch mehrere Hospitationen zu überfordern und möglichst viele Praxen zu beteiligen, wurde eine entsprechende Zuweisung vorgenommen. Praxen im ländlichen Bereich wurden von den Studierenden nur nach verpflichtender Zuteilung besucht und konnten teilweise nicht vermittelt werden, vor allem dann, wenn den Studierenden kein eigenes Fahrzeug zur Verfügung stand. In 2 Fällen sagte die Einrichtung den Kandidatinnen und Kandidaten ab, weil schon Hospitationen anderer Studierender geplant waren.

### Reflexionsbericht

Die Studierenden gaben den Reflexionsbericht zum vorgegebenen Zeitpunkt ab. Die Studierendengruppe von 2021 erzielte einen mittleren Score von 2,9 (Standardabweichung (SD) = 1,1), die Gruppe von 2022 einen Score von 1,5 (SD = 0,6). Die mittleren Scores und die erreichten Punkte für die Kategorien Inhalt, Struktur und Form sind in Tab. 2 dargestellt. Der Unterschied zwischen den Gruppen war statistisch signifikant (*p* < 0,00001). Für alle 3 Bereiche Inhalt, Struktur und Form erzielte die Gruppe von 2022 höhere Punktwerte.

**Table 2:** 

Bewertungskategorien und max. Punktzahl	Studierende 2021 (*n* = 26), durchschnittliche Punktzahl und mittlerer Score	Studierende 2022 (*n* = 25), durchschnittliche Punktzahl und mittlerer Score
Inhalt (max. 10 Punkte)	5,8 (SD = 2,1)	8,3 (SD = 1,1) *
Struktur und Stil (max. 5 Punkte)	2,1 (SD = 1,5)	4,2 (SD = 0,7) *
Form (max. 5 Punkte)	2,9 (SD = 1,2)	4,4 (SD = 0,6) *
Gesamt (max. 20 Punkte)	10,8 (SD = 4,3)	16,9 (SD = 1,9) *
*Mittlerer Score*	*2,9 (SD* *=* *1,1)*	*1,5 (SD* *=* 0,6) *

Zur Berechnung des Scores wurde eine 5‑stufige Skala mit gleichen Intervallen verwendet: Score 1: 17–20 Punkte, Score 2: 13–16 Punkte, Score 3: 9–12 Punkte, Score 4: 5–8 Punkte und Score 5: ≤ 4 Punkte

*SD* Standardabweichung

* Mann-Whitney-U-Test, *p* < 0,05

### Evaluation

Die ausgefüllten Fragebögen wurden 2021 von 22 der 26 Teilnehmenden, 2022 von allen 25 Studierenden abgegeben. Die Bewertung zeigte stabile Ergebnisse über die 2 Jahrgänge. Die mittlere Bewertung aller Items lag für die Gruppe 2021 bei 1,4 (SD = 0,24) und für die Gruppe 2022 bei 1,5 (SD = 0,24). Der Ergebnisse waren statistisch nicht signifikant verschieden (*p* = 0,45). Die mittleren Bewertungen der einzelnen Items sind in Abb. 1 für die fakultären (Abb. 1a) und die fachspezifischen Fragen (Abb. 1b) dargestellt. Für alle Items wurden über beide Jahre überwiegend gute und sehr gute Bewertungen vergeben. Die Anteile sehr guter Bewertungen fielen für die Items „Anregung zum vertiefenden Studium“, „fächerübergreifende Lehre“ und die „Anforderungen der Veranstaltung“ im Vergleich mit den anderen Items geringer aus.

Anmerkungen im Freitext zeigten für 5 Studierende, dass sie den zeitlichen Aufwand als zu hoch empfanden. 3 Studierende empfanden die Zeit zur Hospitation als zu kurz. 4 Studierende wünschten sich in den begleitenden Vorlesungen mehr fächerübergreifende und zahnmedizinspezifische Inhalte. Es wurde vereinzelt angeregt, weitere zahnmedizinische Inhalte in die ersten beiden Semester zu integrieren, um dort vorhandene Freiräume intensiver zu nutzen. Die Werte für die interne Konsistenz (Cronbachs Alpha) waren akzeptabel. Sie betrugen für den universitären Anteil für 2021 und 2022 jeweils 0,71 und 0,68. Für den fachspezifischen Anteil betrugen die Werte entsprechend 0,69 und 0,85.

### Symposium

In den Symposien wurden von beiden Studierendengruppen Präsentationen zu jeweils 5 Themen gehalten. Eine Übersicht über die insgesamt 9 verschiedenen Themen findet sich in der Infobox. Zusätzlich wurde über die Vereinbarkeit von Familie und Beruf sowie Frauen als Zahnmedizinerinnen intensiv diskutiert. An den Symposien nahmen neben Studierenden und Lehrpersonal bis zu 20 Zahnärztinnen und Zahnärzte teil, die sich auch intensiv an den Diskussionen beteiligten.

#### Infobox Themen, die von den Studierenden während des Abschlusssymposiums des Fachs Berufsfelderkundung bearbeitet wurden

- Vergleich von allgemein-zahnmedizinischen und spezialisierten Praxen
- Kommunikation im zahnmedizinischen Team und mit den Patienten
- Motivation, den Beruf zu ergreifen
- Teambildende Maßnahmen und Personalführung
- Rolle des Zahnmediziners als Unternehmer
- Wirtschaftliche Situation zahnmedizinischer Praxen
- Vergleich von selbstständiger zahnmedizinischer Tätigkeit und Tätigkeit im Angestelltenverhältnis
- Verhältnis von Arbeitszeit, Entgelt und persönlicher Zufriedenheit
- Umgang mit Patientengruppen, die besondere Bedürfnisse haben

## Diskussion

Das Fach Berufsfelderkundung stellt eine Neuerung im Curriculum des Studiengangs der Zahnmedizin dar. Das Fach ist zwar in der ÄApprO ebenfalls vorhanden, jedoch ist die dort aufgewandte Zeit erheblich kürzer (1 Tag). Ausgestaltung und Verantwortlichkeit variieren an den unterschiedlichen Standorten erheblich [11]. In einer eigenen, nicht repräsentativen Recherche an mehreren medizinischen Fakultäten konnte dies bestätigt werden. Zum Teil sollen die Studierenden nur für einen Tag hospitieren, lediglich beobachten und keine weiteren Aufgaben erfüllen. An anderen Standorten bekamen die Studierenden Aufgaben und waren aufgefordert, einen Bericht zu ihrer Hospitation anzufertigen.

Im Zeitraum der Einführung der ZApprO fehlte die Zeit, über eine grundlegende Abstimmung zwischen den zahnmedizinischen Ausbildungsstandorten hinauszugehen. Es wurde zwar im Rahmen eines Symposiums des Arbeitskreises für die Weiterentwicklung der Lehre in der Zahnmedizin (AKWLZ) im Juni 2022 in Frankfurt am Main die Ausgestaltung des Faches thematisiert, jedoch waren die Ansätze der Fakultäten auch hier äußerst unterschiedlich. Ebenso waren die Verantwortlichkeiten der zahnmedizinischen Fächer heterogen. Die bisher einzige bekannte wissenschaftliche Untersuchung zur Berufsfelderkundung in der Zahnmedizin wurde als Pilotprojekt am Universitätsklinikum Schleswig-Holstein mit dem Fokus auf Kommunikation durchgeführt [12].

An der Universität des Saarlandes wurde die Entscheidung getroffen, die Berufsfelderkundung vor allem in Praxen des Saarlandes durchzuführen. Die Einbeziehung der niedergelassenen Kolleginnen und Kollegen unter Vermittlung der Ärztekammer, Abteilung Zahnärzte, wurde von Anfang an angestrebt. In Diskussionen im Vorfeld wurde deutlich, dass außeruniversitär tätige Zahnmedizinerinnen und Zahnmediziner bereit sind, studienbegleitende Mentorenrollen [13] einzunehmen. Im Sommer des Jahres 2021 wurden 2 Online-Informationsveranstaltungen angeboten, die dann von 63 Praxen wahrgenommen wurden. Im Saarland gibt es ca. 530 zahnmedizinische Praxen, von denen hier etwa 12 % erreicht werden konnten. Die Ankündigung der Veranstaltungen erfolgte über den Verteiler der Kammer. In den Veranstaltungen wurden Informationen zum Studiengang unter den Bedingungen der ZApprO und insbesondere zur Hospitation und zur Famulatur angeboten. Letztendlich erklärten sich 44 Praxen bereit, Studierende aufzunehmen bzw. zugewiesen zu bekommen. Zusätzlich wurden die Akademie für Zahnärztliche Fortbildung in Karlsruhe sowie niedergelassene Kolleginnen und Kollegen, die über eine außerplanmäßige Professur der Universität des Saarlandes verfügen, außerhalb des Saarlandes einbezogen. Von den Praxen wurde kein systematisches Feedback eingeholt, vereinzelte Rückmeldungen waren jedoch positiv und die Studierenden fühlten sich in den meisten Fällen willkommen. Letzteres wurde durch die Evaluation bestätigt. Insgesamt bestätigt die Stabilität der Verfügbarkeit der Praxen, dass Interesse am Austausch und an der Beteiligung von niedergelassenen Zahnmedizinerinnen und Zahnmedizinern an der Ausbildung der Studierenden besteht. Der Ansatz, dass alle Evaluationen und Reflexionsberichte anonymisiert wurden, könnte ein Faktor gewesen sein, dass es kaum Rücktritte von der Bereitschaft zur Teilnahme durch die Praxen gab. Erfreulich war, dass alle Zahnärztinnen und Zahnärzte auch ihre Bereitschaft zur Teilnahme an der zukünftig stattfindenden Famulatur signalisiert haben. Es wurde allerdings deutlich, dass ländliche Praxen, die mit öffentlichen Verkehrsmitteln nur schwer erreichbar sind, weniger im Fokus der Studierenden standen als Praxen, die am Standort der Medizinischen Fakultät oder an Hauptverkehrsachsen lagen. Es wurde versucht, Studierende, die über ein eigenes Fahrzeug verfügten, gezielt den entfernteren Einrichtungen zuzuordnen. Dieses Vorgehen war mit geringen Abstrichen erfolgreich.

Das Fach Berufsfelderkundung wird an der Universität des Saarlandes im ersten Semester des Curriculums angeboten. Hintergrund war, dass im Sinne eines Constructive Alignment die Erkundung des Berufsfeldes an den Anfang gesetzt werden sollte, um den Studierenden die Möglichkeit zu bieten, eine eventuelle Fehlentscheidung bei der Wahl des Studienfaches frühzeitig zu korrigieren. Gleichzeitig sollte die positive, frühzeitige Begegnung mit dem zahnmedizinischen Team im realen Berufsfeld die Studierenden motivieren und die professionelle Haltung fördern [8]. Die Evaluation bestätigte hier, dass die Studierenden und auch die Zahnmedizinerinnen und Zahnmediziner hoch motiviert beteiligt waren.

An der Universität des Saarlandes wurden die Praktika der Zahnmedizinischen Propädeutik im 3. und 4. Semester eingeordnet. Somit ist bis auf das 2. Semester, das durch die Ausbildung im Fach Anatomie dominiert wird, die Zahnmedizin durchgehend vertreten. In Auswertung der Evaluationen der Berufsfelderkundung ist eine Diskussion entstanden, auch Anteile der Zahnmedizinischen Propädeutik weiter in Richtung des Studienbeginns zu verlegen. Ob dies sinnvoll ist, wird jedoch erst nach Abschluss des ersten Ausbildungsabschnitts nach 4 Semestern abschließend diskutiert werden können.

Inhaltlich beschränkte sich die Berufsfelderkundung nicht auf eine reine Hospitation. Die Studierenden waren angehalten, Erlebtes zu reflektieren und gezielt Erkenntnisse zu gewinnen. Im Sinne einer „hidden agenda“ wurde versucht, erste Grundlagen zur Abfassung wissenschaftlicher Arbeiten und zur Präsentation von Ergebnissen zu vermitteln. Hier zeigte sich, dass es nicht genügte, eine Beschreibung der geforderten Berichtsstruktur anzubieten. Die Ergebnisse der Bewertung der Reflexionsberichte verbesserte sich signifikant, nachdem der zweiten Kohorte ein Best-Practice-Beispiel an die Hand gegeben wurde.

Die Präsentation der Themen auf dem Abschlusssymposium war im Gegensatz dazu von Beginn an durchaus ansprechend. Vermutlich war hier die geforderte Teamarbeit der Schlüssel zu einer hohen Motivation und Qualität. Es wurden etliche Themen bearbeitet, die wichtige Aspekte des Berufsfelds betreffen, wie den Umgang mit Patientinnen und Patienten mit besonderen Bedürfnissen, die Spezialisierung und die Kommunikation im Team und mit den Patientinnen und Patienten.

Die Evaluation durch die Studierenden zeigte in beiden Jahren 2021 und 2022 überwiegend gute und sehr gute Bewertungen. Im Bereich des Wissenstransfers, abgebildet durch die Items „Anregung zum vertiefenden Studium“ und „fächerübergreifende Lehre“, waren die sehr guten Bewertungen geringfügig weniger häufig. Um die Berufsfelderkundung stärker in das Curriculum zu integrieren, wird angestrebt, die fächerübergreifende Vorbereitung der Studierenden zu intensivieren. Dazu sollen die zu diesem frühen Zeitpunkt des Studiums angebotenen Grundlagenfächer Chemie, Biologie und Biophysik hier zumindest punktuell einbezogen werden. Die Anforderungen der Veranstaltung an die Studierenden wurden heterogen bewertet. Positiv zu werten ist, dass die Studierenden die Berufsfelderkundung überwiegend nicht als verlorene Zeit empfinden.

Die Einführung des Fachs Berufsfelderkundung fand unter den Bedingungen der COVID-19-Pandemie in Deutschland statt. Es kam hinzu, dass durch die Anordnung, dass das Fach im ersten Semester stattfinden soll, die Studierenden die zahnmedizinischen Einrichtungen im Herbst und Winter der Jahre 2021/2022 und 2022/2023 aufsuchten. Positiv anzuführen ist, dass trotz der bestehenden Restriktionen die Hospitationen durchgeführt werden konnten. Inwiefern die Ergebnisse der Reflexion und der Evaluation dadurch beeinflusst wurden, kann erst in den kommenden Jahren abgeschätzt werden.

## Fazit

Zusammenfassend kann ausgeführt werden, dass das gewählte Format für das Fach Berufsfelderkundung mit flankierender fachlicher und wissenschaftlicher Begleitung der Hospitation in 2 Jahrgängen erfolgreich umgesetzt werden konnte. Die Studierenden erfuhren grundlegende Einblicke in die Profession und sie erlangten erste Erfahrungen zur Abfassung wissenschaftlich strukturierter Niederschriften sowie zur Gestaltung eines wissenschaftlichen Symposiums. Den Studierenden konnte die Komplexität des Berufsfeldes bewusst gemacht werden. Die zahnmedizinischen Einrichtungen stehen aufgrund positiver Erfahrungen weiter für die Hospitationen der Studierenden zur Verfügung, sodass auch hier eine Verknüpfung der Ausbildung mit der Berufsrealität erreicht werden konnte.
